# Bringing the “self” into focus: conceptualising the role of self-experience for understanding and working with distressing voices

**DOI:** 10.3389/fpsyg.2015.01129

**Published:** 2015-08-07

**Authors:** Sarah F. Fielding-Smith, Mark Hayward, Clara Strauss, David Fowler, Georgie Paulik, Neil Thomas

**Affiliations:** ^1^School of Psychology, University of Sussex, Brighton, UK; ^2^Sussex Partnership NHS Foundation Trust, Hove, UK; ^3^School of Psychology, University of Western Australia, Perth, WA, Australia; ^4^Schizophrenia Research Institute, Darlinghurst, NSW, Australia; ^5^Brain and Psychological Sciences Research Centre, Swinburne University, Melbourne, VIC, Australia; ^6^Monash Alfred Psychiatry Research Centre, The Alfred, Melbourne, VIC, Australia

**Keywords:** self, self-experience, voice hearing, auditory verbal hallucinations, psychosis, schizophrenia, phenomenology, psychological intervention

## Abstract

A primary goal of cognitive behavior therapy for psychosis (CBTp) is to reduce distress and disability, not to change the positive symptoms of psychosis, such as hearing voices. Despite demonstrated associations between beliefs about voices and distress, the effects of CBTp on reducing voice distress are disappointing. Research has begun to explore the role that the psychological construct of “self” (which includes numerous facets such as self-reflection, self-schema and self-concept) might play in causing and maintaining distress and disability in voice hearers. However, attempts to clarify and integrate these different perspectives within the voice hearing literature, or to explore their clinical implications, are still in their infancy. This paper outlines how the self has been conceptualised in the psychosis and CBT literatures, followed by a review of the evidence regarding the proposed role of this construct in the etiology of and adaptation to voice hearing experiences. We go on to discuss some of the specific intervention methods that aim to target these aspects of self-experience and end by identifying key research questions in this area. Notably, we suggest that interventions specifically targeting aspects of self-experience, including self-affection, self-reflection, self-schema and self-concept, may be sufficient to reduce distress and disruption in the context of hearing voices, a suggestion that now requires further empirical investigation.

## Introduction

Voice hearing experiences are common in a range of mental health conditions (Waters et al., 2012a), and often contribute significantly to distress, disability and need for care (Nayani and David, 1996). Cognitive behavioral therapy for psychosis (CBTp) is the recommended first-line psychological treatment for distressing voice hearing experiences occurring in the context of psychotic disorder (American Psychiatric Association, 2004; Royal Australian and New Zealand College of Psychiatrists, 2005; National Collaborating Centre for Mental Health, 2014). When working with people distressed by voice hearing experiences, this approach aims to modify appraisals about the identity, power, knowledge and intent of voices, as these are proposed to determine the degree of distress and interference with functioning experienced by the hearer (Thomas et al., 2014).

Randomized controlled trials (RCTs) are consistent in demonstrating evidence for beneficial but modest effects of CBTp on global measures of voice hearing experiences (van der Gaag et al., 2014). Some RCTs have found evidence for changes in physical, cognitive and behavioral, but not emotional, dimensions of voices (Valmaggia et al., 2005; Peters et al., 2010), a surprising finding given that symptom-related distress is the intended outcome of CBTp. Farhall et al. (2009) suggest that this might be due, in part, to insufficient attention toward targeting voice-related mechanisms in CBTp trials. However, whilst a recent trial targeting a specific type of voice appraisal (voice power beliefs) in people experiencing command hallucinations found a significant reduction in compliance behavior, again, no significant treatment effect was found on voice-related distress (Birchwood et al., 2014).

Given these modest treatment effects, and in particular the limited impact of therapy on voice distress, researchers have suggested that the development of interventions should be further guided by research on processes specific to voice hearing (Thomas et al., 2014). One potential therapeutic approach is suggested by evidence of a range of disturbances in self-experience in people diagnosed with psychosis, including perceived loss of self-coherence, self-identity and self-esteem, amongst other self-related constructs (McCarthy-Jones et al., 2013b). Since voice hearing is one of the defining features of schizophrenia (it has been estimated that approximately 60% of those receiving this diagnosis hear voices; Slade and Bentall, 1988), it is possible that disturbances in self-experience are in some way related to the emergence of and adaptation to voice hearing experiences in (and possibly beyond) psychosis. Indeed, many researchers have posited a central role of self-disturbance in the development of voice hearing experiences in schizophrenia (Sass and Parnas, 2003; Raballo and Larøi, 2011; Larøi et al., 2012; Perona-Garcelán et al., 2015), and cognitive models of psychosis emphasize the role of anomalous self-experiences and beliefs about the self in the nature of and adaptation to voices (Garety et al., 2001; Morrison, 2001; Paulik, 2012).

This evidence suggests that self-experience might represent a viable target for therapeutic intervention in voice hearing. Such a therapeutic focus on self-experience confers several advantages. If disturbances of self-experience do indeed represent the core vulnerability underlying psychotic experiences such as voices, targeting self-experience may have broader effects extending to other symptoms associated with voice hearing, such as paranoia, negative symptoms, depression and anxiety. Furthermore, targeting self-experience may be an alternative, less confronting, means of reducing distress with persons for whom beliefs about voices are maintained with delusional conviction, or who do not wish to engage directly with their voices in therapy. This is also consistent with evolutions of cognitive therapy which see a focus on self-acceptance and a richer understanding of self as an integral part of a person-based approach (Chadwick, 2003, 2006).

However, cognitive behavioral research and intervention to date has typically focused on only two elements of self-experience in voice hearing; negative self-schema and self-esteem, largely neglecting significant advances in the understanding of self-experience from research in phenomenological psychiatry and cognitive and social psychology (García-Montes et al., 2012). Furthermore, the sheer range of concepts embraced by the concept of “self-experience” currently precludes attempts to move beyond this preliminary understanding within the voice hearing literature (Berry and Bucci, 2014). Developing a shared conceptualisation of how these various elements of self-experience relate to one another, and the role that these might play in voice hearing experiences, may help to develop intervention strategies that target these aspects more efficiently. The current paper therefore aims to bring together some of the diverse literature on self-experience, in order to present a conceptual framework for understanding and researching self-experience in voice hearers.

The paper will first outline the current conceptualisation of self-experience in the cognitive behavioral literature, before introducing a framework for understanding the structure of self-experience, based on research in phenomenological psychiatry, cognitive science and social psychology. This framework will be discussed in relation to research findings on disturbed self-experience in psychosis and voice hearing. We will consider a range of potential treatment targets suggested by this framework, and discuss how current intervention strategies may directly or indirectly target these mechanisms. The paper concludes with a discussion of the future research that will be necessary to validate this framework within voice hearers and identify effective intervention strategies to target elements of disturbed self-experience.

## Current Conceptualisations of “self” in CBT for Psychosis

Whilst many researchers within the cognitive behavioral tradition have investigated the relationship between voices and elements of self-experience such as self-schema and self-esteem, Beck, Bentall, Chadwick and colleagues are amongst the few who have attempted to integrate these constructs within a cohesive cognitive framework (Bentall et al., 1994; Beck and Rector, 2003; Bentall, 2003; Chadwick, 2003, 2006; Beck et al., 2008). These authors build on work from cognitive and social psychology in order to describe the content, organization and functioning of the self-concept in people experiencing psychosis.

Social cognitive models propose that the self-concept comprises a collection of abstract and language-based cognitive representations stored in memory which serve to interpret and organize self-relevant actions and experiences (Markus and Wurf, 1987). These self-representations concern various aspects of individuals’ relations with the outside world and with themselves (Higgins, 1987; Beck et al., 2008), including representations of “actual” self (i.e., the specific model of the self that we currently hold in mind), “ideal” self (the self that I would like to be), “ought” self (the self that I ought to be) and “possible” self (the self that I imagine I might become in the future). These representations are proposed to be constructed via continuous reciprocal interaction between the self and the social world (Markus and Wurf, 1987). What results from this process of construction is a dynamic interpretive structure that both derives from and mediates most significant intra- and inter-personal functions, including information processing, affective and behavioral regulation, social perception, and the selection of, and behavior within, social interactions (Markus and Wurf, 1987).

These various self-representations are proposed to be organized within higher-order cognitive structures, which Beck et al. (2008) referred to as schemas. At any one time, only a subset of self-schema are accessed and invoked, depending on both the self-motives being served and the particular demands and affordances of the immediate social situation (Markus and Wurf, 1987). Chadwick (2006) reserves the term schema for the cognitive–affective *experience* of self that we are aware of in any given moment as a particular subset of self-representations become activated. This continually shifting experience of self, which can include associated cognitive, affective and physiological states, has alternatively been referred to as the *working* self-concept (Markus and Wurf, 1987; Bentall, 2003).

Contemporary cognitive models emphasize the significance of recognizing the complex and dynamic nature of the self-concept (Bentall, 2003; Chadwick, 2006). For example, a distinction has recently been made in the psychosis literature between knowledge and evaluative components of the self-concept (Fowler et al., 2006). Whilst the stored schematic representations described previously make up the knowledge component of the self (i.e., what people believe about themselves), the evaluative component comprises constructs such as self-esteem (i.e., how people feel about what they believe about themselves; Maddux and Gosselin, 2012) and self-efficacy beliefs (i.e., what people believe they can do and achieve with their skills and abilities under certain conditions; Maddux and Gosselin, 2012). It has been suggested that the accessibility of, and interactions between, these diverse knowledge-based and evaluative self-representations is as central to functioning as schematic content (Chadwick, 2006). Self-representations will differ in terms of their tendency to become activated or to enter conscious awareness, and different self-representations may be contradictory or poorly integrated, potentially resulting in the actuation of negative emotional states (Bentall, 2003).

These concepts have been explored predominantly within the paranoia and persecutory delusions literature (e.g., Bentall et al., 2001; Freeman et al., 2002; Tiernan et al., 2014). Early cognitive models proposed that persecutory delusions reflect an attributional defense, preventing latent negative self-representations from entering conscious awareness (Bentall et al., 1994, 2001). More recent theories (e.g., Freeman et al., 2002) suggest that paranoia and delusions do not serve a defensive function, but instead build directly upon negative beliefs about the self (e.g., that the self is weak or vulnerable), whereby such beliefs may lead to an experience of oneself as an “easy target” in interpersonal interactions, in turn prompting the anticipation of threat that characterizes persecutory delusions (Salvatore et al., 2012b). In support of the latter theory, empirical work has consistently demonstrated the presence of both negative explicit and implicit self-representations in individuals with paranoia (Tiernan et al., 2014).

In relation to voice hearing specifically, research to date (recently reviewed by Berry and Bucci, 2014) suggests a close link between negative knowledge-based self representations and voice content (Close and Garety, 1998; Barrowclough et al., 2003), appraisals of voice power (Birchwood et al., 2000, 2004; Thomas et al., 2015), and emotional and behavioral responses to voices such as distress (Birchwood et al., 2004; Smith et al., 2006) and compliance (Fox et al., 2004). Whilst some descriptions of the cognitive model suggest a reciprocal relationship between voices and self-beliefs, whereby negative self-schema are suggested to influence both voice content (Beck and Rector, 2003) and appraisals of voices (e.g., Morrison, 2001), and negative voices reinforce and strengthen negative distressing core beliefs about the self (Close and Garety, 1998; Freeman et al., 2002; Chadwick, 2006), the directionality of these effects have yet to be established empirically.

Research into the relationship between the evaluative component of the self-concept and voice hearing experiences has focused predominantly on self-esteem. Recent research has found evidence of an association between low trait self-esteem and negative voice content (Smith et al., 2006), power appraisals (Peters et al., 2012) and distress (Smith et al., 2006), whilst Fannon et al. (2009) found that low self-esteem predicted depression in voice hearers *independently* of voice appraisals. To date, there has been less of a focus on the role of self-efficacy beliefs (another aspect of the evaluative component of the self-concept) in voice hearing, although this is frequently stated as a target for psychological therapy (e.g., Chadwick, 2003; Hayward et al., 2009). However, self-efficacy beliefs have been shown to be an important variable in determining whether voice hearers require psychiatric care (Honig and Romme, 1998), and have been suggested to be undermined by enduring experiences of critical or commanding voices (Rector and Leahy, 2006).

## Bringing the “self” into Focus: A Phenomenologically-informed Framework

These conceptualisations of self within the CBTp literature emphasize the role of stored higher-level representations, evaluations and reflective processes in the experience of self. This is not surprising, since cognitive behavioral approaches are typically reliant on processes of conscious self-reflection to foster insight and facilitate change (Chadwick, 2006). However, it is widely acknowledged that stored knowledge and evaluations are only one aspect of self-experience (Sedikides and Skowronski, 1997; Damasio, 2003), and much more fundamental disturbances of self are also characteristic of psychosis (Moe and Docherty, 2014; Nelson et al., 2014). Indeed, the negative or disordered higher level self-representations which have been described may develop in reaction to more fundamental, pre-reflective disturbances in experience of self (Nelson et al., 2014; Stanghellini and Rosfort, 2015). Specifically, parallel developments in the fields of phenomenological psychiatry and cognitive psychology suggest that these disturbances in self-experience might well: (a) underlie the emergence of and adaptation to voice hearing; (b) contribute to additional distress/disruption in those who hear voices.

Whilst anomalous self-experiences are acknowledged to be an important pre-disposing factor in cognitive models of psychosis (Garety et al., 2001; Paulik, 2012), little attempt has been made to fully integrate these higher and lower-level conceptualisations of self in relation to voice hearing experiences, despite the suggestion that it is the *interaction* between bottom-up and top-town representations and processes that ultimately determines the perception of and adaptation to voice hearing experiences (Waters et al., 2012a; Aleman and Vercammen, 2013). The aim here is therefore to introduce a framework for conceptualising the contribution of various dimensions of self-experience to voice hearing, incorporating insights from phenomenological psychiatry, cognitive, social and developmental psychology.

These literatures present a vast array of overlapping definitions, perspectives and explanations of the self, each with their own associated terminology. Despite this range of perspectives, the majority appear to assume that there is some elementary form of order, structure, or “essence” to our experience (or “sense”) of self, which remains relatively consistent both within and between individuals (Markus and Wurf, 1987; Damasio, 2003; Gallagher and Zahavi, 2012). Furthermore, there appears to be a certain degree of convergence between disciplines around how we might begin to conceptualise this fundamental structure of self.

For the present purpose, self-experience, or sense of self, is defined as the feelings of singularity, coherence, individuality, and unity that define one as a unique and particular human being (Harré, 1998; Damasio, 2003; Prebble et al., 2013). The present framework takes as its basis Parnas’s (2003) description of the structure of the self, which is founded on insights from phenomenological investigations of self-experience within both healthy individuals and those with a diagnosis of schizophrenia (Sass and Parnas, 2003). This framework corresponds closely to those derived independently within the fields of social psychology (e.g., Sedikides and Skowronski, 1997) and cognitive neuroscience (Damasio, 1999), and thus sits on firm empirical ground. This framework posits that there are three inter-dependent levels of self-experience, termed the pre-reflective, reflective, and narrative selves. These will each be described in turn, and evidence for their role in relation to the emergence of and adaptation to voice hearing experiences examined.

### The Pre-reflective Self

According to Parnas (2003), the pre-reflective self (also referred to as minimal, ecological, basic, or proto self, selfhood or self-as-subject; James, 1890; Gibson, 1986; Damasio, 1999; Nelson et al., 2014; Stanghellini and Rosfort, 2015) constitutes the foundational level of selfhood on which other levels of self-experience are built (Nelson et al., 2014) and as such is the basis of various aspects of conscious experience (Nelson et al., 2014a). It is a pre-conceptual, tacit level of selfhood that refers to the implicit sense of being a subject of experience, i.e., a non-inferential first-person awareness that our on-going experiences, thoughts, perceptions, feelings, or actions are our own, accompanied by a sense of immersion in the surrounding world (Parnas, 2003).

A body of phenomenological research provides evidence of disturbances at the level of pre-reflective self-experience in people diagnosed with schizophrenia, which manifest as a range of anomalous subjective experiences (Nelson et al., 2009). These experiences include disturbed presence (disrupted sense of “inhabiting” ones own experience), corporeality (anomalous bodily experiences, such as perceived morphological change or motor disturbances), stream of consciousness (anomalous cognitive processes, such as perceptualization of inner speech or thought, or thought interference), self-demarcation (loss or permeability of self-world boundary, such as confusion between oneself and other people), and existential reorientation (fundamental reorientation with respect to worldview). Sass and Parnas (2003) propose that these basic disturbances in ipseity lie at the core of voice hearing experiences, and may even be a trait marker for psychotic vulnerability (Nelson et al., 2014). In line with this, Kim et al. (2010) have found a significant association between anomalous self-experiences and voice hearing (but not delusions) in patients with schizophrenia, and Raballo and Larøi (2011) present evidence from first-person accounts that anomalous self-experiences could prelude to the development of voice hearing experiences. Varese et al. (2011) have demonstrated using the experience sampling method (ESM) that patients with auditory hallucinations are more vulnerable to dissociative states (an example of disrupted self experience) in response to daily life stress compared to non-hallucinating patients and healthy controls. Furthermore, within the hallucinating patient group, auditory hallucinations were significantly predicted by dissociation, especially under high stress.

Distorted or unstable self-experience is proposed to result from two complementary aspects (Sass, 2014): diminished self-affection (a weakened sense of existing as a subject of awareness or agent of action) and operative hyper-reflexivity (a non-volitional tendency for focal attention to be directed toward processes and phenomena that are normally tacit or implicit). Gallagher and Zahavi (2012) propose that in the case of voice hearing experiences, diminished self-affection reflects a disturbance in the pre-reflective sense of agency (i.e., the sense of being the agent of an action) over inner speech, with ownership of these experiences (i.e., the sense that I am the one who is undergoing these experiences) remaining relatively intact. This proposition is evidenced by the observation that in general, voice hearers remain aware that they, themselves, are hearing voices, rather than somebody else. This disrupted sense of agency, in combination with operative hyper-reflexivity, is suggested to underlie the external attribution of inner dialogue; inner speech becomes the object, rather than the subject, of experience, resulting in the emergence of voices (Gallagher and Zahavi, 2012).

Researchers have recently highlighted a correspondence between these phenomenological disturbances and findings within the neurocognitive literature (Nelson et al., 2014a,b). Specifically, it is suggested that disturbances of self-affection are associated with source monitoring deficits (which result in difficulty identifying self-generated events, and are proposed to result in the misattribution of inner speech; Bentall, 1990), whilst operative hyper-reflexivity correlates with compromised memory-prediction processes (which promote excessive attention to information that is irrelevant or highly familiar, a phenomenon known as “aberrant salience”; Kapur, 2003). Indeed, errors on source monitoring tasks are one of the most robust findings in relation to hallucinations; for example, Waters et al. (2012b) have presented meta-analytic evidence that source monitoring/self-recognition is impaired in patients with schizophrenia, and particularly in those who hear voices. However, it is unclear whether these neurocognitive disturbances constitute the neural underpinnings of disturbed self-affection, or whether they lie downstream from a more core alteration of basic self-experience (Sass, 2014).

If a disorder of basic self-experience were primary, this would help to explain phenomenological differences between inner speech and voices (Parnas, 2003). Among the most characteristic features of voices in psychosis are so-called “Schneiderian” hallucinations (Daalman et al., 2011); a voice describing the patient’s on-going behavior or experience, or two or more voices discussing the patient in the third person. Such third-person commentary is not a common feature of typical inner speech (Langdon et al., 2009), creating problems for theories which suggest that voices simply reflect misattributed inner speech. Parnas suggests that the processes of diminished self-affection and operative hyper-reflexivity result in a transformation in the *nature* of inner speech; experiences of self-alienation result in a shift away from inner speech referring to self-as-subject (e.g., “I need to make breakfast and get the kids to school in 20 min”) toward self-as-object (e.g., “he’s going into the kitchen……now he’s opening the fridge…”). This “higher-level” inner speech (which reflects the self-consciousness that generates self-alienation) is proposed to form the content of Schneiderian hallucinations (Parnas, 2003). However, whilst it might be expected from this account that voice hearers should experience less “typical” inner speech than non-voice hearers, a recent phenomenological study of inner speech found that this is not the case (Langdon et al., 2009). It is possible that inner speech referring to the hearer in the second or third person is simply more likely than typical inner speech to be misattributed; however, the potential reasons for this are not specified within either the phenomenological or source monitoring accounts of voice hearing.

### The Reflective Self

The reflective self, also referred to as the core self (Damasio, 1999), self-as-object (James, 1890) or objectified self (Sedikides and Skowronski, 1997) refers to a primitive cognitive representation of the self as the invariant and persisting subject pole of experience and action (Parnas, 2003). This representation is accessed via reflective consciousness (i.e., the capacity of an organism to become the object of its own attention). This reflexive, or metacognitive, capacity occurs at a substantially more explicit or conscious level than the elementary sense of ipseity provided by the pre-reflective self (Sedikides and Skowronski, 1997), but is both derivative and dependent upon it (Parnas, 2003). Research in individuals with a diagnosis of schizophrenia has found evidence for disturbances in the reflexive consciousness that is central to the functioning of the reflective self.

Phenomenological theorists such as Sass and Parnas (2003) suggest that the lack of automatic identification of experiences as one’s own (which results from diminished affection and operative hyper-reflexivity at the level of the pre-reflective self—see The Pre-reflective Self), may subsequently prompt more reflective forms of hyper-reflexivity (Nelson et al., 2014b). Such “hyper-reflection” (or increased conscious self-reflection) might occur as an in-the-moment reaction (e.g., the sense that this “voice” did not originate in me leads to reflection about its potential origins), but it may also turn into something more habitual, such as increased attentional focus on particular elements of experience (Nelson et al., 2014b). It has been proposed that these consequential and compensatory processes may serve to further a sense of alienation and diminished self-affection (Parnas, 2003), potentially reinforced by the “objectifying” content of Schneiderian hallucinations. This notion of hyper-reflection demonstrates clear parallels with self-focused attention, which has been at the center of much cognitive behavioral research (García-Montes et al., 2012). Thus, in support of this phenomenological account, there is evidence that high levels of self-focused attention are associated with the experience of, or predisposition toward, hallucinations in both patients (Morrison and Haddock, 1997; Ensum and Morrison, 2003) and non-clinical participants (Allen et al., 2005). Furthermore, Perona-Garcelán et al. (2008) demonstrated a positive correlation between self-focused attention and dissociative experiences in patients with hallucinations, but not in patients with psychoses who have never experienced hallucinations.

Contemporary dialogical theories agree that processes such as hyper-reflexivity and hyper-reflection contribute to basic problems with self-recognition of thoughts and emotions (Lysaker et al., 2012), but they posit that these issues are further compounded by additional metacognitive deficits, which can result in an inability to see one’s own thoughts processes as subjective and fallible (Dimaggio and Lysaker, 2015). Specifically, Lysaker and Dimaggio (2014) propose a deficit in *synthetic* metacognition; the capacity to recognize and integrate basic elements of experience into complex representations of self and others, and to adopt a superordinate meta-position which allows one to question one’s beliefs and use one’s knowledge to respond to psychosocial challenges. It can easily be imagined how difficulties in adopting a superordinate stance might even serve to promote further hyper-reflection around individual elements of experience, further exacerbating anomalous experiences such as voice hearing. In line with this view, people with a diagnosis of schizophrenia have been found to experience trait like deficits in the ability to form and use complex metacognitive self-representations (Lysaker and Dimaggio, 2014), and reduced metacognitive capacity to comprehend one’s own mental states has been found to predict increased severity of auditory hallucinations in people with psychosis (Lysaker et al., 2005).

### The Narrative Self

The narrative self (Nelson et al., 2014), also referred to as the symbolic (Sedikides and Skowronski, 1997; Chadwick, 2006), social (Parnas, 2003) or autobiographical self (Damasio, 1999), dynamic self-concept (Markus and Wurf, 1987) or personhood (Stanghellini and Rosfort, 2015) refers to the collection of abstract and language-based cognitive representations stored in memory which serve to interpret and organize self-relevant actions and experiences (Markus and Wurf, 1987). This is the level at which the majority of cognitive behavioral research in psychosis has been conducted (see Current Conceptualisations of “self” in CBT for Psychosis). This level of self is widely understood to presuppose the pre-reflective and reflective selves (Sedikides and Skowronski, 1997; Damasio, 1999; Nelson et al., 2014), and to rely on the reflective self, in addition to a capacity for encoding and retrieving episodic–autobiographical memories (Gallagher, 2003; Prebble et al., 2013), for the development, consolidation, and refinement of its content, structure and functions (Sedikides and Skowronski, 1997; Nelson et al., 2014).

As we have seen, cognitive models discuss how constructs associated with the narrative self, such as low self-esteem and negative self-beliefs, might influence the way in which anomalous experiences, such as voice hearing, are interpreted (Waters et al., 2012a). However, a recent meta-synthesis of qualitative accounts of the lived experience of psychosis highlights the reported loss of additional aspects associated with the narrative self, including self-coherence and self-identity (McCarthy-Jones et al., 2013b). There has been much less emphasis within the cognitive literature on how these higher level self-constructs might relate to psychotic experiences such as voice hearing.

Salvatore et al. (2012b) have recently attempted to integrate the cognitive and phenomenological conceptions of the role self-experience in paranoia, suggesting that negative beliefs about self as weak or vulnerable may in fact emerge from, or be compounded by, the pre-reflective self-disturbances discussed previously, resulting in an experience of self as “ontologically vulnerable” (Salvatore et al., 2012b). Stanghellini and Rosfort (2015) elaborate on this idea, suggesting that “self-disorder, being at the core of the vulnerability to schizophrenia, is refracted through the prism of the person’s background of values and beliefs that determine what things and events in the world mean for us” (p. 4). Whilst this idea demonstrates clear parallels with the cognitive notion that the meaning and impact of anomalous experiences are mediated by beliefs (Chadwick et al., 1996; Garety et al., 2001), the phenomenological conception goes further to suggest that anomalous pre-reflective self-experience may represent the foundations on which both psychotic experiences and self-identity are reciprocally constructed and appraised. Thinking about voice hearing experiences specifically, experiencing the self as “ontologically vulnerable” could serve to reinforce appraisals of voice power, proposed by cognitive models to be an important factor in determining voice-related distress (Birchwood et al., 2000, 2004; Gilbert et al., 2001).

Similarly, dialogical theorists have elaborated significantly on suggestions by Bentall (2003) and Chadwick (2006) in the cognitive literature that the accessibility and dynamic interactions between different self-representations is as important as the content of these representations in determining functional adaptation to anomalous experiences (Lysaker and Lysaker, 2002, 2004). Dimaggio et al. (2010) cite evidence from the clinical and social psychology literature suggesting that psychological health and social adaptation might depend on; (i) the existance of a sufficient number of self-representations (or “positions”), enabling a range of strategies for coping with the complexities of social life; (ii) the ability to have conscious access to self-representations that are context-relevant; (iii) the ability of different self-representations to be aware of each other and engage in a “dialogue” (achieved via inner speech) during inter- and intra-personal exchanges; (iv) the ability of the person to adopt a superordinate point of view, or metaposition, allowing an adaptive decision or resolution to be achieved via the coordination of and negotiation between different self-representations. In the context of psychosis, it is suggested that the aforementioned deficits in synthetic metacognition (see The Reflective Self) may result in difficulties at each of these levels (Dimaggio et al., 2010; Lysaker et al., 2012; Lysaker and Dimaggio, 2014) resulting in the disturbances in self-coherence and self-identity described in the psychosis literature, including an overly negative, disordered or “barren” self- concept (Lysaker et al., 2012).

This theory predicts that individuals with a diagnosis of psychosis might demonstrate disturbances in terms of; (i) self-concept clarity (i.e., the degree to which one’s beliefs about one’s attributes are clear, confidently held and cognitively accessible; Stinson et al., 2008); (ii) self complexity (i.e., the number and distinctiveness of accessible self-representations; Linville, 1987); (iii) the complexity and/or phenomenology of inner speech. In support of these suggestions, in non-clinical populations, low self-concept clarity, in combination with a tendency toward experiences of aberant salience (i.e., in which stimuli that ordinarily would not seem significant become much more salient and important; Kapur, 2003), is associated with an increased risk of psychotic-like experiences (Cicero et al., 2013). In individuals with a diagnosis of schizophrenia, low self-concept clarity has been found to predict higher levels of positive symptoms, lower subjective quality of life and increased risk of perceived exposure to stigmatization (Weinberg et al., 2012; Noyman-Veksler et al., 2013). In relation to voice hearing specifically, Bell and Wittkowski (2009) found evidence for reduced *positive* self complexity in voice hearers, and an association between higher positive self complexity and increased psychological wellbeing. However, at odds with the predictions of early dialogical theories, a recent study found that the phenomenology of inner speech in voice hearers does not differ significantly from that of non-clinical participants (Langdon et al., 2009).

Perona-Garcelán et al. (2015) have suggested that, rather than voice hearing experiences simply serving to disrupt the flow of internal conversation (as suggested by Lysaker and Lysaker, 2005), voices might in fact *themselves be a product of the dissociation of different self-positions*. In this view, increased self-focused attention/hyper-reflection on particular self-positions lead to a loss of metacognitive perspective, whereby these self-positions are no longer experienced as “belonging” to the owner. Instead, certain self-positions are experienced as interpersonal “others,” prompting individuals to interact with voices in a similar manner to their interactions with others in the social environment. Support for this view includes evidence of an increased tendency toward dissociative experiences in voice hearers (Perona-Garcelán et al., 2008); findings that voice hearing experiences share some of the pragmatic properties of communication between people (Leudar et al., 1997); and finally, evidence that the relationship between the person and his or her voices is similar to the relationship between individuals in a social context (Hayward, 2003). However, many questions still remain as to the nature of the relationship between voice hearing experiences, inner dialogue and disturbed higher-level self-concept.

## Targeting Self-experience in Voice Hearers

Researchers have recently highlighted the need to identify novel targets for psychological therapy for distressing voices, given the modest effects of CBT-based treatment approaches on both global measures of voice hearing experiences, and outcomes related to emotional dimensions of voices such as voice-related distress (Thomas et al., 2014). The evidence outlined in the previous section suggests that disturbances in self-experience occurring at the pre-reflective, reflective and narrative levels are likely to be intimately connected with the experience of voice hearing, in terms of both etiology and functional and emotional adaptation, and as such, may represent viable therapeutic targets in attempts to provide relief from distressing voices.

It is worth noting that whilst CBT-based approaches have tended to consider distress primarily as a consequence of hearing voices, to be addressed by intervening with how voices are appraised and responded to, it is likely that the relationship between voice hearing, voice-related distress and emotional distress more generally is complex, reciprocal, and difficult to partition. It is possible that the limited impact of CBTp on voice-related distress might reflect this complex relationship; for example, the cognitive and behavioral mechanisms targeted by CBTp may well lie “downstream” of distress in the causal pathway, or some latent third variable may underlie the emergence of both voice hearing experiences and psychological distress. Considering the contribution of self experience to this picture may be a means of conceptualising broader ways in which voice experience interacts with distress, rather than it merely being an adaptational endpoint.

For example, it is likely that disrupted self-experience may in itself be distressing for many voice hearers; the sense of ontological vulnerability associated with pre-reflective self disturbance may contribute to a basic feeling of unease with oneself, additional stress whilst navigating social interactions with other people, and reduced resilience in the face of critical voice content. Further, distress and emotional arousal could directly serve to influence pre-reflective self-experience, perhaps by disrupting neurocognitive processes that ordinarily facilitate a stable, situated sense of self.

As such, targeting aspects of self-experience might be expected to have effects that extend beyond symptom-related distress, to include many other facets of psychological distress. Indeed, many current interventions for schizophrenia, psychosis, and distressing voices incorporate methods that explicitly aim to target some of these aspects of self-experience. Given the recent emphasis within the literature on developing focused intervention methods that aim to modify specific therapeutic targets and processes (e.g., Thomas et al., 2014), we will now briefly consider what therapeutic methods may be suited to targeting self-experience, with reference to potential applications of components of current interventions that have been described in the literature.

### Pre-reflective Self

At the pre-reflective level of self-hood, interventions are required that target the phenomenological disturbances proposed to underlie anomalous self-experiences, voice hearing and other psychotic symptoms, such as disrupted self-affection and hyper-reflexivity (see The Pre-reflective Self).

#### Targeting Disrupted Self-affection and Hyper-reflexivity

Two main approaches have been described which might enhance basic sense of self (Nelson et al., 2014b). There is preliminary evidence that reconstructive mind-body exercises (used within body-oriented psychotherapy; Röhricht and Priebe, 2006) might help to reduce basic self-disturbances in outpatients with a diagnosis of schizophrenia, re-establishing a coherent sense of embodied self, self-directed and vitalized social interaction and emotional expressiveness (Röhricht et al., 2009). These movement-based exercises enable participants to directly engage with their bodies in order to recognize and articulate unusual reflections suggestive of disembodiment, somatic disintegration and other abnormal bodily sensations. Whilst it might be expected that the restored ipseity achieved by this approach might translate into a reduction in voice hearing experiences and associated distress, this possibility has yet to be explored.

A second approach is to correct neurocognitive processes which may be contributing to disrupted self-affection and hyper-reflexivity. There is a demonstrated association between these phenomenological disturbances and deficits in attention, sensory processing and source monitoring, and it has been suggested that neurocognitive remediation therapies targeting these mechanisms may be beneficial ([Bibr B96][Bibr B97]; Postmes et al., 2014). Of course, the success of these approaches will depend crucially upon the nature of the relationship between these processes and self-disturbance; it is currently unclear whether these neurocognitive correlates lie up- or down-stream from alterations of basic self-experience (Sass, 2014). If neurocognitive disturbances are causal in the genesis of anomalous self-experience, and ipseity disturbances are related to the emergence of voice hearing experience in the way proposed by phenomenologists, cognitive remediation might be expected to fundamentally alter the voice hearing experiences of those undergoing treatment. However, again, the success of these approaches in restoring basic self-experience, and any associated impact on voices, has not yet been evaluated.

### Reflective Self

Interventions can alternatively aim to target disturbances of self consciousness proposed to underlie or reinforce disrupted self-experience, such as hyper-reflection and impaired metacognitive processing (see The Reflective Self).

#### Minimizing Hyper-reflection

Results of phenomenological investigations suggest that the sense of loss of self in schizophrenia involves compromised first- and second- person awareness, leaving affected individuals able only to view themselves from “the outside” (Stanghellini and Lysaker, 2007). From the perspective of phenomenological researchers such as Nelson and Sass (2009), the aim of psychological therapy is to enable patients to “re-inhabit,” rather than objectify or externalize, their experience, and to assist a patient in beginning to recapture the ability to engage in first- then second-person experiences (Stanghellini and Lysaker, 2007). This approach warns that traditional cognitive approaches, particularly those which challenge the negative content of thoughts and schemas, might exacerbate hyper-reflective processes, leading to increased focus on the self as the object, rather than the subject, of experience (Pérez-Álvarez et al., 2011).

Stanghellini and Lysaker (2007) outline four general principles of phenomenonologically-informed therapy; (i) highlighting disturbances of intersubjectivity; (ii) aiming at achieving a shared partnership, (iii) focusing on the here-and-now, you-and-I relationship, and (iv) pointing to shared meaningfulness. This approach focuses on the promotion of the experience of self in the second person as a necessary first step to change. It is suggested that the primary task of the therapist is promoting the construction of micro-narratives focussed on real world situations, by consistently reflecting back a second-person perspective on the person’s experiences (e.g., “*you* are feeling bad”; “*you* feel that you can’t do anything right”). The aim of these micro-narratives is to assist in dialogically co-constructing a shared second-person view of the patients experiences, improving the person’s capacity to sense experiences as his or her own, reflect upon them, and take an intentional stance over them, re-establishing disrupted immediate connectedness between the self and the present situation (Stanghellini and Lysaker, 2007).

Whilst the phenomenological conception warns against hyper-reflective processes promoted by traditional cognitive therapy, it additionally endorses strategies that stimulate a mindful awareness and acceptance of subjective experiences, particularly when these allow exploration of personal meaning (Pérez-Álvarez et al., 2011). Such approaches may be particularly helpful in addressing the contribution of “Schneidarian” voice content toward the exacerbation of hyper-reflective processes. Other recommended treatment approaches include those which encourage gradual engagement in activities and social interaction, thereby promoting a sense of immersion in present activity (Nelson and Sass, 2009). Such approaches are seen as beneficial not only in reducing hyper-reflection, but also in re-establishing normal ipseity at a pre-reflective level, as they promote a “forgetting” of the self as an objective focus of awareness, so that a more foundational sense of oneself as a subjective perspective on the world can be grounded (Nelson and Sass, 2009). Social and occupational engagement are emphasized by a number of exisiting interventions, for example Social Recovery Focused Cognitive Therapy (Fowler et al., 2009), Acceptance and Commitment Therapy (Gaudiano et al., 2010) and Person Based Cognitive Therapy (Chadwick, 2006), and may be particularly helpful for voice hearers given evidence that the intensity of voice hearing decreases during active engagement in leisure activity (Delespaul et al., 2002).

#### Enhancing Metacognitive Awareness

Lysaker and Dimaggio (2014) suggest that interventions should aim to help patients to recapture the ability to notice and integrate the different elements that make up mental activities, and to become capable of increasingly complex metacognitive acts. Lysaker et al. (2011) suggest that the first step of a metacognition oriented therapy (MOT) should be to assess a clients capacity for self-reflectivity; for example, determining whether they are able to notice that they have thoughts in their head, recognize these thoughts as their own, distinguish between different cognitive operations, and/or notice and identify their own feelings. This is seen to be a critical first stage of therapy, since discussing feelings or attempting to challenge cognitions when these cannot be recognized is assumed to be counterproductive, perhaps constibuting to frustration and distress on the part of both client and therapist.

The second step of therapy is to offer interventions appropriate to the patient’s current level of metacognitive functioning. Lysaker et al. (2011) offer a variety of suggestions for interventions appropriate to different levels of metacognitive capacity for self-reflectivity, all of which use a consultative process in which the therapist adopts a reflective, questioning stance in order to create a space in which clients internal experiences can be openly thought about (Bargenquast and Schweitzer, 2013). For example, if a client is currently unable to determine whether there are thoughts in their own mind, an appropriate approach might be to provide reflections on the specific thoughts that a client may currently be experiencing, based on what the client chooses to talk about. As with the phenomenological approach, reflections in the second-person are thought to be useful since these reflections implicitly treat clients as participants in their lives, recognizing that they are more than passive observers (Lysaker and Lysaker, 2011). If a client is able to recognize and articulate different thoughts as they arise, the focus of therapy would shift toward discussing and reflecting back these specific mental operations, and supporting the client to build these fragments into a more complex, coherent and personalized narrative.

At higher levels of metacognitive functioning, interventions may involve supporting clients to think about their or other’s thinking and feelings in an ever more complex manner, by asking questions such as “what was that like for you?” or “how do you think that made × feel?” (Bargenquast and Schweitzer, 2013). At the highest levels of self-reflectivity, the therapist aims to work alongside the client to consider the fallibility of their thinking, take a step back from firmly held ideas, and begin to consider alternative world views. Again, points of internal debate can be brought into focus by providing reflections based on ongoing discussion, for example; it seems that there is a part of you that believes ×, but also another part of you that has some doubts” (Lysaker et al., 2011).

Whilst the effect of MOT on reducing distress and disruption in relation to voice hearing experiences has yet to be directly assessed, reductions in voice frequency have been observed in individual case studies (Salvatore et al., 2012a), and it is likely that such an approach would facilitate interventions aimed at restoring processes of narrative self-construction (described in see Targeting Higher-level Constructs: Self Esteem and Self Concept). Furthermore, evidence from the delusions literature suggests that similar metacognitive training approaches can be used to promote insight into the cognitive biases serving to maintain unusual beliefs (Kumar et al., 2014), which may serve to augment cognitive behavioral interventions for voice hearing.

### Narrative Self

Interventions operating at the narrative level can aim to target lower-level constructs, such as the content, meaning, accessibility and impact of positive and negative self schema, and/or to influence the way in which these lower-level self representations interact to produce higher-level constructs such as self-esteem, self-concept and identity.

#### Modifying the Content, Meaning, and Impact of Negative Self-schemas

Traditional cognitive approaches attempt to modify self-schematic content. This starts with the collaborative formulation of negative self-schema (Padesky, 1994; Chadwick, 2006) and is followed by the use of Socratic techniques to review and evaluate evidence (naturally occuring evidence as well as evidence gathered through behavioral experiments) as to the accuracy of negative self-schemas (Padesky, 1993) and/or to strengthen positive self beliefs, such as beliefs about personal power and status relative to voices (Trower et al., 2004).

However, modifying negative schematic content might not always be possible, as this content may be stored in memory in a way that is not easily amenable to change (Hayes, 2004; Brewin, 2006; Longmore and Worrell, 2007). An alternative approach with the narrative self is to modify meta-cognitive beliefs about negative schematic experiences using a combination of behavioral, mindfulness- and acceptance-based methods—that is, shifting away from the meta-cognitive belief that self-schema are literal truths about self and toward a recognition of their contradictory, transient and non-literal nature. Paul Chadwick’s “two chair method” aims to highlight the transient and non-literal nature of self-schematic experiences through promoting metacognitive distancing from these experiences (Chadwick, 2003, 2006). Adapted mindfulness practice, metaphors and experiential exercises can be used to further facilitate re-appraisal of the status, rather than the content, of negative self-schema (Chadwick, 2006; Thomas et al., 2013).

In addition to modifying meta-cognitive beliefs about self-schema, habituation to schematic distress (as opposed to changing content) has also been proposed. Strategies demonstrated to be effective for voice hearers include shame attacking exercises (drawn from Rational Emotive Therapy) which create opportunities through repeated voluntary exposure to feel and accept how schematic distress can be tolerated, and will habituate (Chadwick, 2006).

Finally, approaches that enhance self-compassion are showing promise for people experiencing psychosis in reducing distress and social marginalization (Braehler et al., 2013), with benefits also found specifically for people hearing voices (Mayhew and Gilbert, 2008). Compassion-focused therapy (CFT; Gilbert, 2009) does not directly aim to alter self-schematic content but rather attempts to enhance self-soothing and to dampen activation of the threat system through compassion-focused exercises. Similarly, one of the mechanisms of mindfulness-based approaches appears to be through enhanced self-compassion (Gu et al., 2015) and this may be due to the emphasis on acceptance and non-judgement, including of self-schematic experiences, in these approaches. Approaches that enhance self-compassion therefore have potential to reduce the impact of negative self-schematic experiences as the person learns to self-sooth and to accept and not judge these experiences.

Given the demonstrated links between negative self-schema and voices (Close and Garety, 1998; Birchwood et al., 2000, 2004; Barrowclough et al., 2003; Fox et al., 2004; Smith et al., 2006), such treatment approaches might be expected to influence the content, appraisal and impact of voice hearing experiences, in addition to addressing any independent contribution of negative self-schema to wellbeing.

#### Enhancing the Accessibility and Content of Positive Self-schemas

Many researchers have argued that work on negative self-schema should be complimented by attempts to enhance awareness of and strengthen conviction in positive self-schema (e.g., Chadwick, 2006). Furthermore, demonstrated associations between positive self-schema and positive beliefs about voices (Thomas et al., 2015) are consistent with the suggestion that attempts to enhance positive self-schemas might have an impact on voice appraisals, although the causal direction of these effects is not yet known and is likely to be complicated.

Traditional CBT methods such as evidence gathering (historical, in current daily life and through behavioral tasks) and mental rehearsal can be used to strengthen conviction in and accessibility of positive schema (Tarrier, 2001; Hall and Tarrier, 2003), including self-efficacy beliefs such as “I have some control even when voices are around” (Morrison et al., 2004; Chadwick, 2006).

Other techniques aim to increase the accessibility of positive self-schema. For example, the two chair method used within PBCT allows patients to articulate and “embody” positive schema whilst momentarily distancing themselves from negative schematic experiences (Chadwick, 2003). A similar approach is used within Competitive Memory Training (COMET; van der Gaag et al., 2012), whereby patients are asked to repeatedly recollect and relive past experiences in which they felt successful or competent. This “re-living” involves imagining these positive experiences whilst adopting a congruent posture and facial expression and (sub) vocalizing a congruent positive self-statement (van der Gaag et al., 2012).

A final approach is to promote the development of new positive schematic content. Since the development and maintenance of self-schema are dependent upon interpersonal experience (Markus and Wurf, 1987), behavioral approaches which promote social and occupational engagement (e.g., Hodgekins and Fowler, 2010) or target assertiveness and communication skills (Hayward et al., 2009) could all potentially contribute to the development of new positive schematic content.

#### Targeting Higher-level Constructs: Self Esteem and Self Concept

Whilst attempts have been made to directly target higher-level concepts such as self esteem (Lecomte et al., 1999), it has been suggested these constructs may be more helpfully viewed as treatment outcomes (or intermediaries between target and outcome), rather than targets for intervention *per se* (Hayes et al., 2013). It has been proposed that such “global” or “aggregate” concepts subsume a number of lower-level constructs, which might represent more viable targets for intervention (Kazdin, 2007; Hayes et al., 2013). In line with this idea, the most robust improvements in self-esteem in those with a psychosis diagnosis have been demonstrated in an intervention targeting the accessibility of positive self-schema (Hall and Tarrier, 2003). Similarly, improvements in self-concept clarity and complexity are most likely to be achieved by targeting the processes via which lower-level self-representations interact and integrate to form complex and consciously accessible higher-level self-constructs (Dimaggio et al., 2010). Since this process is likely to rely upon reflexive capacities, restoration of synthetic metacognitive awareness using the techniques outlined previously (see Enhancing Metacognitive Awareness) is proposed to provide a platform for further narrative self-construction (Chadwick, 2006; Lysaker and Dimaggio, 2014).

Beyond the enhancement of metacognitive capacities, the process of self-construction can be facilitated using a number of techniques. The two-chair method provides an opportunity for clients to explore the interactions between newly elaborated positive and negative self-schema, allowing their eventual integration within a new metacognitive model of self as complex and changing (Chadwick, 2006; Dannahy et al., 2011). Alternatively, narrative metacognitive therapies place an emphasis on enhancing the client’s capacity for dialogical self-narration (Bargenquast and Schweitzer, 2013). This approach aims to first enhance the client’s awareness of themselves as storyteller by encouraging narration of their experience in-session, and exploring and expanding upon story fragments expressed by the client. As in metacognition oriented therapy, techniques such as reflection and questioning are used to facilitate the discovery of undeveloped or suppressed self-aspects, and to develop complexity in a client’s narratives. Therapy additionally aims to target problems with dialogue between different self-positions, by collaboratively exploring and connecting material discussed within the current and previous sessions, highlighting the existence of alternative and potentially conflicting self facets, and modeling functional interpersonal dialogue in-session (Lysaker and Lysaker, 2011). Finally, addressing the content and meaning of voice hearing experiences within a broader life narrative is likely to be an important element of therapy, a perspective that is emphasized by approaches associated with the Hearing Voices Movement (e.g., Corstens et al., 2008; Longden et al., 2012).

## Key Research Questions

Future research should aim to validate and refine this model within voice hearers in order to improve intervention strategies. A number of key research questions can be identified.

### What is the Relationship Between Self- and Voice-experience?

Whilst there is good evidence for disturbances occurring at all three levels of self in people with psychosis, the extent to which these various disturbances in self-experience are related specifically to (i) voice hearing experiences and (ii) voice-related distress and disruption is currently unclear. In order to determine the specific role of disrupted self-experience in voice hearing experiences, future research should first seek to compare the prevalence and nature of these experiences in people with a diagnosis of psychosis who hear, or do not hear, voices.

This research should start with an in-depth exploration of the phenomenology of self- and voice-experience (McCarthy-Jones et al., 2013a), and subsequently extend beyond an assessment of the role of negative self-schema and low trait self-esteem, to include both lower- and higher-level aspects of self-experience such as disturbed ipseity, metacognitive awareness and various dimensions of self-concept (Tiernan et al., 2014). In doing so, it will be important to return to the social psychology literature in order to clarify the conceptual and theoretical relationship between different constructs such as implicit and explicit self-esteem, self-concept complexity, clarity and flexibility. One reason for doing so is the evidence of a close relationship between self-concept clarity and state self-esteem (Campbell, 1990; Nezlek and Plesko, 2001), which might have important implications for how to target low self-esteem in voice hearers.

Understanding of the role of these experiences in voice-related distress and disruption will be aided by an exploration of the nature and prevalence of disturbed self-experience across the voice hearing continuum (for example, in voice hearers with diagnoses other than psychosis, and in those without any need for care). Not only can such an approach be helpful toward identifying key targets for psychological therapy, but given the suggestion that a disturbance of the basic sense of self is a phenotypic trait marker of psychotic vulnerability, might also inform future strategies for early intervention (Nelson et al., 2009).

### What is the Direction of any Relationship Between Self- and Voice-experience?

The cross-sectional research outlined above will determine whether disturbances in self-experience are a reliable trait marker of distressing voice hearing experiences, but such approaches say little about (a) the temporal association between these experiences; (b) the directionality of this relationship. Whilst disrupted self-experience is likely to represent a worthy treatment target in its own right, as it can in itself lead to distress and disruption to quality of life, such interventions will have limited impact on voice hearing experiences or associated distress if changes in self-experience are a consequence, rather than a cause of voices.

The temporal association between voice hearing and disrupted self-experience can be assessed using longitudinal approaches that compare the nature of self-experience in; (i) current and past voice hearers, (ii) people within and outside of voice hearing episodes. However, such approaches are still reliant on trait measures of self-experience and voices, and there is good evidence that both voices and self-experiences are likely to vary within-person (i.e., they are state variables; Nezlek and Plesko, 2001; Thewissen et al., 2011). Therefore, of potentially greater relevance to the development of effective psychological interventions for distressing voices is whether various disturbances in self-experience are associated with, and indeed predictive of, the onset of voices during the course of daily life.

Micro-longitudinal momentary assessment approaches such as the ESM (Csikszentmihalyi and Larson, 1987; Oorschot et al., 2009) can shed light on the dynamic relationship between voices, self-experience, and contextual factors (i.e., potential triggers), providing an indication of directionality. McCarthy-Jones et al. (2013a) suggest that ESM could be used to assess the locus of attention of voice-hearers immediately preceding AVHs, potentially revealing whether these experiences are indeed characterized by hyper-reflective processes as suggested by Sass (2003). Such an approach could additionally be used to determine the daily dynamics of the association between voice hearing experiences and (i) altered states of pre-reflective self-awareness (such as dissociation) and (ii) negative self- and social-appraisals at the level of the narrative self. ESM could also be used to shed light on the extent to which disrupted self-experience may contribute to distress and dysfunction *independently* of voice hearing experiences. Given evidence that low self-esteem predicts depression independently of voice appraisals (Fannon et al., 2009), this is likely to be an important consideration when working with voice hearers.

In addition to establishing the temporal relationship between self- and voice-hearing experiences, it will be important to investigate their developmental relationship. Determining the developmental pathways of voice hearing experiences can shed light on potential treatment targets at different stages during development, in addition to having implications for early identification and intervention for voice hearers who are at greater risk of developing a future need for care. Raballo and Larøi (2011) have provided evidence from first-person accounts that anomalous self-experiences could prelude to the development of distressing voices. Furthermore, evidence of a link between both voices and dissociation (a disruption to pre-reflective self-experience) and early traumatic experiences (Varese et al., 2012) hints toward a potential common developmental origin. Prospective epidemiological studies can shed further light on whether disturbances in self-experience precede voices developmentally (or vice versa), and the relationship between these experiences and environmental and demographic factors.

### What Mechanisms Underlie the Relationship Between Self- and Voice-experience?

Future research should additionally seek to determine the nature of the cognitive processes and neurocognitive mechanisms underlying operations occurring at different levels of selfhood. For example, it will be important to determine the nature of the neurocognitive processes underlying diminished self-affection and operative hyper-reflexivity, and of disturbances at the level of the reflective self. Such research can determine the degree to which these phenomena are influenced by context, or are neurologically determined, and thus shed light on potential targets for neurocognitive remediation therapies, which have been demonstrated to be effective in improving global cognition, functioning and symptoms in individuals with a diagnosis of schizophrenia (Wykes et al., 2011). Sass (2014) suggests that psychological manipulations of self-experience (e.g., using meditative techniques or introspection) might help to determine associations with symptoms, neurocognitive performance, and neural correlates in healthy individuals and voice hearers. Such experimental findings could be triangulated with real-time quantitative data obtained using ESM approaches (Eisenberger et al., 2007), in order to identify the neurocognitive correlates of disturbed self-experience during daily life.

A further question is how operations at these different levels of self might interact in the experience of voice hearing. Whilst it is helpful to conceptualise voice hearing experiences in relation to processes occurring at the three levels of selfhood described previously, it is important to emphasize that in reality there are no clear boundaries between these levels, and all interact to produce a dynamic experience of self (Sedikides and Skowronski, 1997). Such an interaction between operations occurring at the different levels of self is consistent with recent neurocognitive models of voice hearing (Waters et al., 2012a; Aleman and Vercammen, 2013), which propose that auditory hallucinations are caused by a blend of distorted input from bottom-up sensory information and aberrant top-down factors (including prior knowledge and memories, perceptual expectations, and mental imagery).

Preliminary evidence suggests that self-representations at the level of narrative self might have a “top-down” influence upon the interpretation of anomalous self-experiences occurring at a pre-reflective level (Synofzik et al., 2008; Cicero et al., 2013), and likewise, it has been suggested that disturbances occurring at the pre-reflective and reflective levels of self-hood contribute fundamentally to the formation of idiosyncratic beliefs at the level of the narrative self (Nelson et al., 2014; Stanghellini and Rosfort, 2015). Both anomalous self-experiences and higher-level self-narratives will additionally motivate behaviors (such as avoidance) which may serve to reinforce both lower- and higher-level self disturbance. Empirical investigation of these ideas will serve to further enrich psychological models of voice hearing.

### How Can Self-experience be Targeted in Therapy?

Whilst many current interventions for schizophrenia, psychosis, and distressing voices incorporate methods that explicitly aim to target aspects of self-experience, few studies have investigated; (a) whether these multicomponent interventions are effective in modifying self-related constructs; (b) whether such changes mediate therapeutic gains; (c) which specific therapeutic elements are responsible for these changes.

These are important questions for future research, given recent calls to improve the scientific basis of interventions for distressing voices (Thomas et al., 2014). One approach is to incorporate measurement of self-related treatment targets and mechanisms within the context of RCTs of interventions such as CBTp. However, with self-experience representing one of many targets, and without standardization of the extent to which this is addressed during therapy, this is likely to provide a relatively indirect test.

Research in this field would be aided by the use of dismantling approaches to determine the impact of existing techniques within multi-component interventions (Kazdin, 2007), and interventionist-causal approaches (Kendler and Campbell, 2009; Freeman, 2011), in which specific aspects of self-experience are targeted experimentally in order to determine whether changes in voice experience and its impact can be produced. Such targeted proof-of-concept intervention studies can provide an early indication of which targets are most amenable to change prior to full-scale RCTs (Hayes et al., 2013). However, these approaches require attention to the best operationalization and measurement of outcomes, both in terms of the aspects of self that are being targeted and how to conceptualise and measure voice-related outcomes (Thomas et al., 2014).

## Conclusion

This paper has introduced a framework for conceptualising the role of self-experience in voice hearing. Whilst there is emerging evidence for associations between various aspects of disturbed self-experience in voice hearing, and preliminary attempts to target some elements of self-experience in interventions for voices, further research is required to understand the directionality of this relationship, and its potential for therapeutic intervention. Multi-disciplinary approaches will be central to developing our understanding of the role of self-experience in distressing voices.

### Conflict of Interest Statement

The authors declare that the research was conducted in the absence of any commercial or financial relationships that could be construed as a potential conflict of interest.
